# DFT design of PO_4_@C_60_ nanohybrid unveils superelectrophilic molecular metamorphosis with molecular dynamics exhibiting therapeutic potential against p53 cancer mutant Y220C

**DOI:** 10.1039/d6ra01727d

**Published:** 2026-07-02

**Authors:** Ryan John Laverne, Sudheesh K. Shukla, Ashok Kumar Mishra

**Affiliations:** a Department of Physics, Dr. Shakuntala Misra National Rehabilitation University Lucknow Uttar Pradesh–226017 India akmishra@dsmnru.ac.in akmishra2k5@gmail.com; b Department of Biosciences, Graphic Era (Deemed to be University) Dehradun Uttarakhand–248002 India; c Department of Chemical Sciences, University of Johannesburg, Doornfontein Campus P.O. Box 17011 Johannesburg 2028 South Africa

## Abstract

We report the quantum-chemical design of an endohedral PO_4_@C_60_ (POC) nanohybrid, optimized using DFT-ωB97X-D/6-31g(d), cross-validated *via* the CAM-B3LYP/6-31g(d) functional-basis set combination. POC exhibits a global formation energy of −6.33 eV, undergoing a spontaneous endohedral molecular metamorphosis. QTAIM, RDG, ELF, and NBO analyses confirm that the complex is stabilized through the interplay of intricate non-covalent interactions and internal cage oxidation. The nanostructure exhibits a HOMO–LUMO gap of 5.5 eV, suggesting good chemical reactivity in the reaction medium. Its MESP surface characterizes POC as a superelectrophilic anion with a highly negative, smoothly delocalized surface suitable for interactions with cationic and polar protein environments. The therapeutic rescue of the thermolabile p53–Y220C mutant remains a pivotal goal in precision oncology. Comparative molecular docking reveals that POC exhibits a strong binding affinity of −11.5 kcal mol^−1^, outperforming the docking scores of pristine C_60_ and reported stabilizers MB710 and Rezatapopt. To validate these findings, two independent 100 ns MD simulations of the protein–POC complex were performed, which exhibit stable MD metrics, indicating that POC does not perturb the global fold acting as a pharmacological chaperone. Thermodynamic evaluation using MM/PBSA and MM/GBSA yields binding free energies of −38.78 kcal mol^−1^ and −43.73 kcal mol^−1^, respectively, with an acceptable *in-silico* toxicity profile. These insights position this superelectrophilic nanohybrid as a promising candidate for restoring the pro-apoptotic function of the p53 tumor suppressor.

## Abstract 2

We report the quantum-chemical design of an endohedral PO_4_@C_60_ (POC) nanohybrid, optimized using DFT-ωB97X-D/6-31g(d), cross-validated *via* the CAM-B3LYP/6–31g(d) functional-basis set combination.

## Graphical abstract

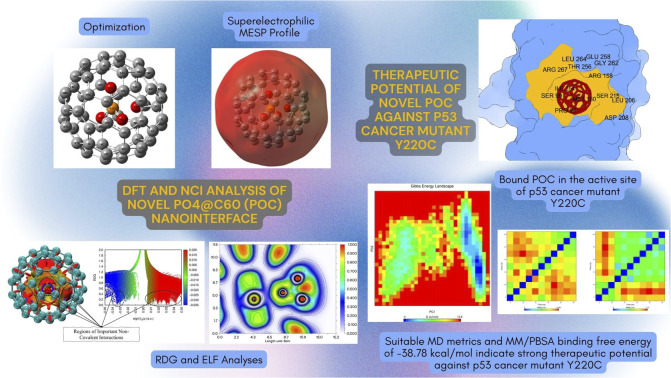

## 1 Introduction

Carbon-based nanomaterials, including fullerenes, carbon nanotubes and graphene, have emerged as fundamental building blocks in materials science, driving transformative advances across electronics, energy storage and nanomedicine due to their unique structural, electronic and chemical properties.^1–3^ Among these, C_60_ buckminsterfullerene stands as the archetypal molecular cage, composed of 60 sp^2^-hybridized carbon atoms arranged in a truncated icosahedral geometry with an internal cavity of approximately 7 Å in diameter.^4^ This highly symmetric spherical structure, featuring a seamless network of pentagonal and hexagonal rings, offers an exceptional nanoscale container capable of encapsulating atoms, ions, and small molecules.^5–7^ However, synthesizing such complexes poses significant experimental challenges owing to the carbon cage's robustness and the stringent conditions required for guest insertion.

Studies on endohedral fullerenes highlight how the C_60_ cage creates a unique, confined environment that stabilizes encapsulated species through strong host–guest interactions and electron transfer mechanisms. While prior studies have reported the encapsulation of small molecules^8,9^ inside C_60_ cages, the incorporation of polyatomic ions such as phosphate (PO_4_^3−^) remains comparatively unexplored, especially concerning the structural and electronic consequences of such endohedral encapsulation. The present investigation demonstrates a structural metamorphosis induced by the partial intra-cage dissociation of an oxygen atom from the phosphate moiety, which permanently oxidizes the internal fullerene shell. This internal confinement of the polyatomic species subsequently prompts a spontaneous reconstruction of the host–guest interface. Fundamentally, molecular metamorphosis represents a profound structural reorganization wherein the chemical system alters its topological arrangement to transition into a distinct, thermodynamically stable minimum.^10,11^ For the present study, the PM6 semiempirical method^12^ has been employed for preliminary charge estimation, followed by rigorous quantum mechanical (QM) computations using density functional theory (DFT)^13,14^ to investigate complexation energy, Mulliken charge distribution, frontier molecular orbital (FMO) analysis, and molecular electrostatic potential (MESP) surface for the endohedral phosphate–fullerene hybrid nanostructure (POC). DFT is a reliable quantum-chemical method that typically yields results in good agreement with experimental data.^15–20^ Non-covalent interactions (NCI) are revealed by reduced density gradient (RDG),^21,22^ quantum theory of atoms in molecules (QTAIM)^23–26^ and electron localization function (ELF)^27^ analyses.

Beyond materials science, pristine C_60_ nanoparticles have emerged as effective modulators of apoptosis due to their remarkable antioxidant properties, which attenuate oxidative stress-induced cellular damage.^28–30^ This biomedical relevance is particularly significant in cancer research, where restoring the stability of mutant tumor suppressor proteins, such as p53, often regarded as the guardian of the genome, remains a compelling therapeutic approach. Among the various cancer-associated mutations, the Y220C variant, the most common, introduces a surface cavity in p53 that compromises its structural stability, even though the mutation site is distant from the DNA-binding domain and does not significantly disrupt the overall protein fold.^31,32^ While wild-type p53 displays only moderate conformational stability, the Y220C mutant readily unfolds under physiological conditions, thereby impairing p53 signaling and promoting tumorigenesis.^33^ Small molecules that bind within the Y220C cavity have been shown to prevent this unfolding and stabilize the mutant protein, as supported by multiple bioinformatics and structural studies.^34,35^

Recent studies further indicate that the p53–Y220C mutation exhibits immunogenic properties, making it an appealing target for emerging immunotherapeutic strategies.^36,37^ Therapeutically, MDM2 and MDMX, key negative regulators of wild-type p53, have been targeted clinically to reactivate p53 function;^38,39^ however, MDM2/MDMX inhibitors demonstrate efficacy in only about half of cancer cases,^40^ highlighting the need for alternative or complementary approaches, such as ligands that directly stabilize mutant p53 proteins, like Y220C, to restore tumor suppressor activity and improve therapeutic outcomes. Although fullerenes have been explored for cancer-related therapy,^41^ to the best of our knowledge, no prior studies have examined the interaction of either pristine C_60_ or the novel POC with the p53 cancer mutant Y220C.

In the present study, the molecular docking approach^42^ has been implemented to study the interactions of POC and pristine C_60_ against the aforementioned cancer mutant. Since POC exhibited a stronger binding affinity relative to pristine C_60_ and the clinical stabilizers MB710 and Rezatapopt,^34,43^ to ascertain further stability, two independent molecular dynamics (MD) simulations^44^ of the docked protein–POC complex were performed to assess interaction dynamics. Analyses include root mean square deviation (RMSD), root mean square fluctuations (RMSF), principal component analysis (PCA), solvent-accessible surface area (SASA) of free and bound ligand, along with ligand burial percentage in the protein cavity. The overall binding free energy was estimated employing molecular mechanics Poisson–Boltzmann surface area (MM/PBSA)^45^ and molecular mechanics generalized Born surface area (MM/GBSA)^46^ models.

This work presents a comprehensive computational investigation combining DFT, RDG, and QTAIM analyses to examine phosphate (PO_4_^3−^) encapsulation and internal bonding within the C_60_ cage. We further explore POC's potential as a stabilizing nanomaterial against p53–Y220C mutation, using MM, MD, and energy computations to predict binding affinity and dynamic behavior in biological environments. These findings advance fundamental understanding of reactive endohedral functionalization in carbon nanocages and open avenues for designing multifunctional nanomaterials in nanotechnology, biomedicine, and cancer therapeutics.

## 2 Methodology

### 2.1 Design and optimization

The POC molecule was initially constructed using GaussView, followed by geometry optimization and electronic property calculations using the Gaussian 09^47^ software suite. Preliminary optimization and charge estimation were carried out with the semiempirical PM6 method,^12^ providing an efficient starting point for subsequent higher-level computations. Thereafter, Density Functional Theory (DFT) calculations were performed using the long-range-corrected hybrid functional ωB97X-D,^48^ which incorporates Grimme's empirical dispersion model (D2).^49^ To ensure the physical robustness of our thermodynamic and electronic claims, key calculations were subsequently cross-validated using a second long-range-corrected functional, CAM-B3LYP.^50^ The 6-31G(d) basis set,^51^ a Pople-type double-ζ basis, was employed for all DFT computations, offering an excellent compromise between computational cost and accuracy. This basis is well known to yield reliable geometries and energetics that are consistent with experimental data for large molecular systems. Comparable computational strategies have been successfully employed by Silva *et al.* in their theoretical investigation of fullerene–carbamazepine interactions in aqueous environments,^52^ thereby supporting the reliability and robustness of the present methodological framework. The individual chemical bonds and the energies associated with lone–pair electrons, which govern the stability and reactivity of the POC nanohybrid, are elucidated by NBO analysis.^53–55^ To probe the internal electronic architecture, the stabilization energy *E*^2^ related to electron delocalization is computed for each donor NBO(i) and acceptor NBO (j).^56,57^

To provide a rigorous assessment of the binding affinity and thermodynamic stability of the POC nanohybrid, electronic energies were calculated at the ωB97X-D/6-31G(d) and the CAM-B3LYP/6-31G(d) levels of theory. The global formation energy for the POC has been calculated by subtracting the energies of the host and guest moieties from the resulting complex, *i.e.*

1 *E*_form_ = *E*_complex_ − (*E*_host_ + *E*_guest_)

The interaction energy (*E*_int_) has also been determined at the same levels of theory, using a fragment-based approach, where the complex was partitioned into fragment 1 (C60) and fragment 2 (PO_4_^1−^). To account for the artificial overestimation of binding affinity, Basis Set Superposition Error (BSSE) was corrected using the Counterpoise (CP) method.^58^ The BSSE-corrected interaction energy is calculated as follows:

2 *E*_int_ = *E*^BSSE^_complex_ − (*E*_fragment1_ + *E*_fragment2_)

### 2.2 QTAIM, RDG and ELF analyses

To investigate the nature of the chemical bonding and non-covalent interactions within the POC complex, topological analyses based on the Quantum Theory of Atoms in Molecules (QTAIM), the Reduced Density Gradient (RDG) method, and the Electron Localization Function (ELF) were performed.^59^ The QTAIM and RDG approaches utilize the evaluation of electron density (*ρ*) and the second eigenvalue (*λ*2) of the electron density Hessian matrix to characterize Bond Critical Points (BCPs) and map intermolecular interactions. Simultaneously, ELF analysis was employed to map regions of electron localization, to characterize the electronic architecture and to confirm the QTAIM and RDG analyses. All wavefunction-based computations were executed using the Multiwfn program,^60,61^ an efficient wavefunction analysis tool. Visualization of isosurfaces and molecular interaction plots was achieved using Visual Molecular Dynamics (VMD) version 2.0.0.^62^

### 2.3 Protein–ligand interaction mapping

The interaction of POC and pristine C_60_ with the Y220 mutation of the p53 tumor suppressor protein was initially screened using a molecular docking approach implemented through the AutoDock Vina program.^63,64^ The protein structure of the p53 cancer mutant Y220C was obtained from the RCSB Protein Data Bank^65^ with PDB ID: 5O1G.^34^ Standard docking protocols were employed to identify optimal binding poses. Standard docking protocol results revealed the best docking poses for both structures, which were visualized using the UCSF ChimeraX^66^ program.

The top-ranked docking conformations served as starting structures for molecular dynamics (MD) simulations using GROMACS, a widely used biomolecular simulation package.^67,68^ The AMBER99SB-ILDN force field^69^ was employed to describe the protein and system interactions, while ligand topologies were generated using the ACPYPE server based on the Antechamber protocol,^70^ with the General AMBER Force Field 2 (GAFF2) implemented for parameterization.^71^ The simulation system was neutralized with Na+ and Cl− ions at a physiological concentration of 0.15 M. To maintain physiological conditions, temperature was controlled at 310 K using a velocity-rescale thermostat, and pressure was stabilized at 1 bar using the Parrinello–Rahman barostat. Two independent 100 ns MD simulations were performed to ensure methodological robustness. Equilibration steps included canonical ensemble (NVT) dynamics to stabilize the temperature, followed by isothermal–isobaric ensemble (NPT) dynamics to achieve the desired system density, using the leap-frog integrator. The first simulation employed 3 ns each of NVT and NPT equilibration, while the second applied 5 ns for both phases. The system was centered and subjected to periodic boundary conditions in all dimensions to emulate an infinite bulk environment. Periodic boundary conditions were initially applied, with corrections to prevent artificial jumps of molecules across the box boundaries. The system was then centered within the simulation box to ensure consistent positioning of all atoms. Finally, overall translational and rotational motions of the protein were removed from the trajectory to facilitate proper alignment and analysis, while preserving molecular flexibility during the simulation.

### 2.4 MM/GBSA and MM/PBSA calculations

Using gmx_MMPBSA v1.6.4,^72^ the binding energies of the protein–ligand complex have been calculated. To ensure equilibrated sampling, both Generalized Born solvent accessible surface area (GBSA) and Poisson–Boltzmann solvent accessible surface area (PBSA) models were implemented for the last 30 ns of the molecular dynamics trajectory. Also, to replicate near physiological conditions, calculations were performed at a temperature of 310 K. Δ*G*_bind_, *i.e.* the estimated free energy of the ligand–protein receptor complex, for both the MM/GBSA and MM/PBSA approaches, is predicted by the program package using the equation:^73^

3 Δ*G*_bind_ = *G*^complex^ − (*G*^complex^ + *G*^ligind^)

Where the average free energy for complex, receptor and protein is calculated as:

4 *G*^*x*^ = *E*_MM_^*x*^ + *G*_solvation_^*x*^ − TS

Here, *x* represents the entity (*i.e.* protein, ligand, or the complex), *E*_MM_ corresponds to the intermolecular energy evaluated in vacuum or gas, *G*_solvation_ corresponds to the solvation free energy in implicit solvent, and TS is the configurational entropy term.

5 Δ*G*_bind_ = *E*^complex^_MM_ − *E*^receptor^_MM_ − *E*^ligand^_MM_ + *G*^complex^_solvation_ − *G*^receptor^_solvation_ − *G*^ligand^_solvation_ − *T*ΔS

6 Δ*G*_bind_ = Δ*G*_gas_ + Δ*G*_solvation_ − *T*Δ*S*

where,

7 〈Δ*G*_gas_〉 = 〈*E*^complex^_MM_〉 − 〈*E*^receptor^_MM_〉 − 〈*E*^ligand^_MM_〉

8 〈Δ*G*_solvation_〉 = 〈*G*^complex^_solvation_〉 − 〈*G*^receptor^_solvation_〉 − 〈*G*^ligand^_solvation_〉

Δ*G*_gas_ represents the gas phase intermolecular energy difference, and Δ*G*_solvation_ is the difference of implicit–solvent free energies between the complex and the separated partners. *T*Δ*S* is the configurational energy due to the change in entropy. The PB model rigorously solves the Poisson–Boltzmann equation numerically, resulting in more accurate polar solvation energies than the GB model, which is much faster because it uses analytical approximations.

### 2.5 Toxicity analysis

Evaluating the toxicological profile of a novel nanohybrid is a critical prerequisite for its advancement as a therapeutic candidate. To ascertain the preliminary safety profile of the POC nanohybrid and its viability for biological applications, an *in silico* toxicity assessment was performed using the Protox 3.0 webserver.^74,75^

## 3 Results and discussion

### 3.1 Structural parameters and frequency analysis

**Fig. 1:**
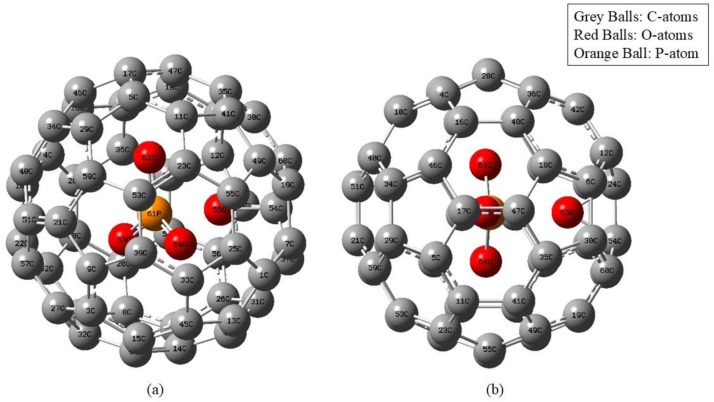
The DFT optimized Guest (PO_4_) – Host (C_60_) Complex at the ωB97X-D/6-31G(d) level of theory (a) side view and (b) top view with atom numbering.

The initial screening for the overall charge of the endohedral heterofullerene (PO_4_@C_60_) was performed using the semi-empirical PM6 method. Geometry optimizations were attempted for multiple charge states, including neutral (0), anionic (−1), dianionic (−2) and trianionic (−3) systems to identify the most stable electronic configuration. The preliminary screening identified the anionic state (−1) to exhibit the lowest total energy, establishing PO_4_@C_60_^1−^ as the thermodynamically preferred configuration, suggesting that the destabilizing effects of confining a trivalent phosphate ion are mitigated by charge reduction. Such an observation aligns with previous DFT studies on similar systems, which report that anion-encapsulated fullerenes behave as large, diffuse anions.^76^ This optimized structure of the most stable PO_4_@C_60_^1−^ complex served as input for reoptimization at the DFT ωB97X-D/6-31G(d) level of theory. The DFT predicted ground-state geometry of this complex is shown in Fig. 1.

A significant structural transformation is observed upon the internalization of a phosphate ion within the C_60_ cage. The initially tetrahedral PO_4_^3−^ ion undergoes partial fragmentation, resulting in the dissociation of one covalent bond between P61 and O65. A molecular metamorphosis is observed as the O65 atom forms covalent bonds with the C54 and C24 atoms, thus generating a covalent-bonded PO_3_ unit within the fullerene cavity, with the O65 oxygen atom oxidizing the fullerene cage. The structural parameters of this computationally designed POC complex are shown in Supplementary Information Table S1.

**Fig. 2:**
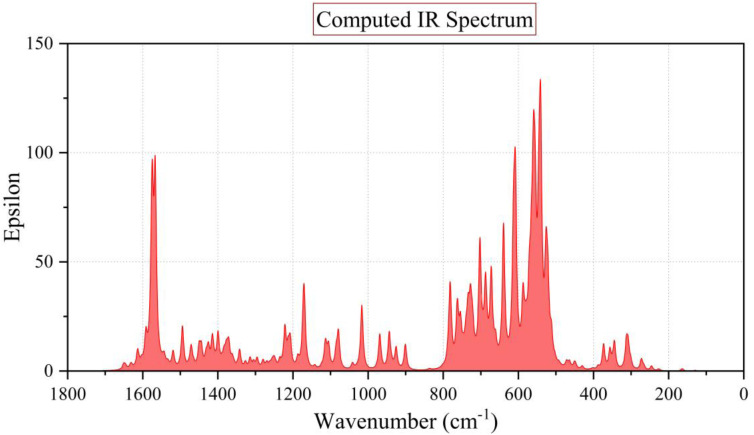
Computed IR Spectrum for POC using DFT at the ωB97X-D/6-31G(d) level of theory.

The structural integrity and thermodynamic stability of the PO_4_@C_60_^1−^ hybrid (POC) have been validated through vibrational frequency analysis at the same level of theory. The absence of imaginary frequencies confirms that the optimized geometry of POC corresponds to a true local minimum on the potential energy surface. 189 active modes of vibration are observed, displayed in Supplementary Information Table S2, consistent with the (3N–6) vibrational degrees of freedom expected for a non-linear molecule.^77^ The IR spectrum yielding from the vibrational frequency analysis for POC is shown in Fig. 2.

### 3.2 Mechanistic pathway and recovery time

The formation of the POC hybrid proceeds *via* a spontaneous two-stage mechanism, involving charge redistribution followed by structural reorganization. Attributed to the fundamental electronic instability of PO_4_^3−^ in non-solvated environments,^78^ DFT calculations performed in the present study also demonstrate that PO_4_^3−^ is more unstable than PO_4_^1−^ because a notably lower energy for PO_4_^1−^ has been obtained than that for PO_4_^3−^ (Table 1). This is consistent with reported finding for monoanionic phosphate species to be intrinsically more stable in isolation.^79^ Following this electronic stabilization, the monoanion spontaneously undergoes a direct structural metamorphosis within the C_60_ cavity, wherein the O65 atom partially dissociates from the P61 atom and forms two covalent bonds with the C_60_ cage, as depicted in Fig. 1. This cage oxidizing rearrangement is consistent with the reported results on fullerene oxygen covalent bonds.^80,81^

Global formation, BSSE-corrected interaction, and estimated electronic relaxation energies computed at DFT-ωB97X-D/6-31G(d) and DFT-CAM-B3LYP/6-31G(d) levels of theory

**Table:** 

**Global formation energy**				
**Energy value at the DFT level of theory**	**Complex (POC) (in au)**	**Guest (PO**_**4**_^**3**^ **^−^) (in au)**	**Host (C**_**60**_ **) (in au)**	**Global formation energy** ***E***_**form**_ **=** ***E***_**C**_ **–** ***E***_**G**_ **–** ***E***_**H**_
ωB97X-D/6-31G(d)	*E*_C_ = −2927.392520	*E*_G_ = −641.703937	*E*_H_ = −2285.455702	−0.23288 au = −6.336 eV
CAM-B3LYP/6-31G(d)	*E*_C_ = −2926.866429	*E*_G_ = −641.721653	*E*_H_ = −2284.944921	−0.19986 au = −5.438 eV

**Table:** 

**BSSE-corrected interaction energy**				
**Energy value at the DFT level of theory**	**Complex (POC) (in au)**	**Guest (PO**_**4**_^**3**^ **^−^) (in au)**	**Host (C**_**60**_ **) (in au)**	**BSSE-corrected interaction energy *E***_**int**_ **= *E***_**C1**_−***E***_**F1**_−***E***_**F2**_
ωB97X-D/6-31 G(d)	*E*_C1_ = −2927.350629	*E*_F1_ = −642.153715	*E*_F2_ = −2285.455702	0.258786 au = +7.042 eV
CAM-B3LYP/6-31 G(d)	*E*_C1_ = −2926.817421	*E*_F1_ = −642.172381	*E*_H_ = −2284.944921	0.299881 au = +8.160 eV

**Table:** 

**Electronic relaxation energy**	
**Energy value at the DFT level of theory**	**Estimated electronic relaxation energy** ***E***_**relax**_ **=** ***E***_**form**_ **–** ***E***_**int**_
ωB97X-D/6-31G(d)	−13.378 eV
CAM-B3LYP/6-31G(d)	−13.598 eV

The calculated energetic and thermodynamic parameters for this encapsulation process are consolidated in Table 2. The thermodynamic stability of this system has been evaluated through an energy decomposition scheme, where the electronic relaxation energy (*E*_relax_) is derived from the difference between the global formation energy (*E*_form_) and the BSSE−corrected interaction energy (*E*_int_). The process yields a highly exothermic global formation energy of −6.336 eV, despite a significant endothermic steric penalty of +7.042 eV incurred by confining the phosphate guest within the cavity. This steric resistance is effectively counterbalanced by a substantial electronic relaxation energy of −13.378 eV released during the PO_4_^3−^ to PO_4_^1−^ transition inside the cage. This stabilization energy accounts for the electronic rearrangement of the trapped phosphate. Since this substantial energy release exceeds the structural rearrangement cost of +7.042 eV, it provides a sufficient thermodynamic driving force that facilitates the spontaneous endohedral encapsulation. Notably, this electronic relaxation energy exceeds the analogous vacuum transition of PO_4_^3−^ to PO_4_^1−^, showing an energy of −12.239 eV (comparing the energies of PO_4_^3−^ and PO_4_^1−^ in Table 1), suggesting that the cage confinement facilitates the electronic transition event. Consequently, the overall structural rearrangement remains globally stabilized and thermodynamically favorable.

**Table 2:** Topological Parameters at AIM Critical Points

Bond	CP	*ρ*	∇^2^*ρ*	*G*	*V*	*H*	−[*G*/*V*]
O62–C17	1	0.0402	0.2018	0.0466	−0.0427	0.0039	1.091
C52–O63	2	0.0350	0.1723	0.0396	−0.0361	0.0035	1.097
C38–O63	3	0.0339	0.1631	0.0374	−0.0341	0.0034	1.097
C58–O63	4	0.0336	0.1642	0.0375	−0.0340	0.0035	1.103
C20–O63	5	0.0304	0.1489	0.0337	−0.0301	0.0036	1.119
C33–O64	6	0.0350	0.1723	0.0396	−0.0361	0.0035	1.097
O64–C25	7	0.0336	0.1643	0.0376	−0.0340	0.0035	1.106
C45–O64	8	0.0339	0.1631	0.0374	−0.0340	0.0034	1.10
C1–O64	9	0.0304	0.1489	0.0337	−0.0301	0.0036	1.12
P61–O65	10	0.0618	0.1114	0.0492	−0.0706	−0.0214	0.697

Unlike traditional endohedral complexes, which are characterized by non-covalently trapped guests,^8,9^ the architecture of POC is fundamentally distinct, highlighting a paradigm shift from existing literature. The oxygen atom from the phosphate moiety partially dissociates from the phosphate atom and forms covalent bonds directly with the cage carbons (discussed in Section 3.4). To ensure methodological robustness, the thermodynamic parameters are cross-validated using the DFT-CAM-B3LYP/6-31g(d) functional-basis set combination (Table 1). The good agreement between these methods supports the thermodynamic analysis, demonstrating that the electronic relaxation energy drives a robust transformation into the formation of POC.

Furthermore, as an approximate descriptor of persistence of the encapsulated phosphate moiety within the fullerene cage, the recovery time (*t*_rec_) of the host–guest system has been evaluated.^82^ The recovery time dictates the likelihood of guest desorption and is expressed as:9
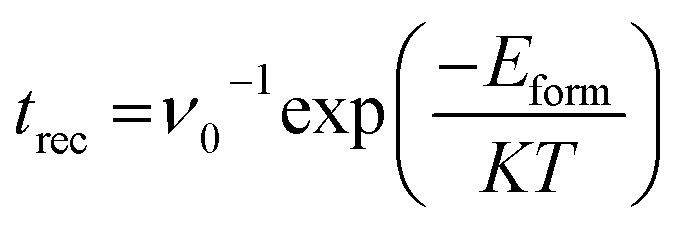
where *ν*_0_ is the attempt frequency (assumed as 10^12^ Hz), *K* is the Boltzmann constant, and *T* is the ambient temperature (300 K). Utilizing the global formation energy, the calculated recovery time for the encapsulated phosphate is approximately 10^87^ years. This large recovery time suggests that the encapsulation and subsequent partial fragmentation of the phosphate ion is an exceptionally persistent structural state that remains thermodynamically stable and highly persistent under ambient conditions, rather than acting as a transiently adsorbed guest. The internal functionalization results in a permanently bonded, structurally stable nanohybrid.

### 3.3 Mulliken charge distribution

**Fig. 3:**
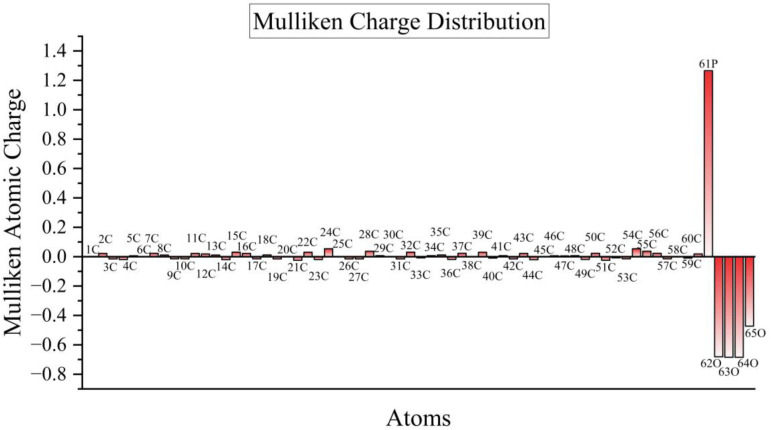
Mulliken charge analysis for novel POC using DFT at the ωB97X-D/6-31 G(d) level of theory.

The PO_3_ fragment remains localized within the oxidized fullerene (C_60_O) structure, resulting in a stable PO_4_@C_60_^−1^ derivative, as suggested by the formation energy calculation. Mulliken charge analysis provides valuable insights into the electronic distribution related to the vibrational characteristics of the host–guest system,^83–85^ shown in Fig. 3. Notably, the phosphorus atom confined within the fullerene carries the highest positive charge of approximately 1.27, while the oxygen atoms exhibit substantial negative charges ranging from −0.47 to −0.68. The PO_3_ segment as a whole carries a total charge of −0.8 in the full complex, indicating that it effectively contributes the −1 charge, with the oxidized fullerene remaining nearly neutral. Although the majority of carbon atoms in the fullerene display minimal charges, it is noteworthy that the carbons directly bonded to the oxygen moiety (C24 and C54) are slightly more positive, each holding a charge of 0.05. This distinct charge redistribution arising from the endohedral functionalization suggests a unique electronic environment across the designed POC.

### 3.4 Non-covalent interactions analysis

**Fig. 4:**
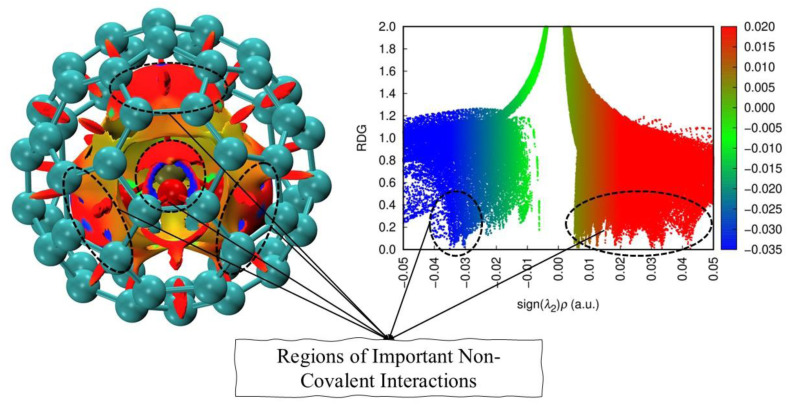
RDG isosurfaces and scatter graphs for novel POC with red, green, and blue colored regions indicating repulsive, van der Waals interactions and strong attractive non-covalent interactions.

RDG and QTAIM have been employed to elucidate the nature of non-covalent interactions within the PO_4_@C_60_^−1^ nanocomplex. The RDG isosurface, mapped onto the molecular geometry, reveals the spatial distribution of weak interactions that stabilize this host–guest system, as shown in Fig. 4. Notably, compact blue regions on the isosurface indicate strong, attractive interactions within the phosphate-functionalized endohedral fullerene, green domains correspond to van der Waals contacts, and red regions signify repulsive steric effects, enabling straightforward visual discrimination of interaction types within the nanocomplex.

**Fig. 5:**
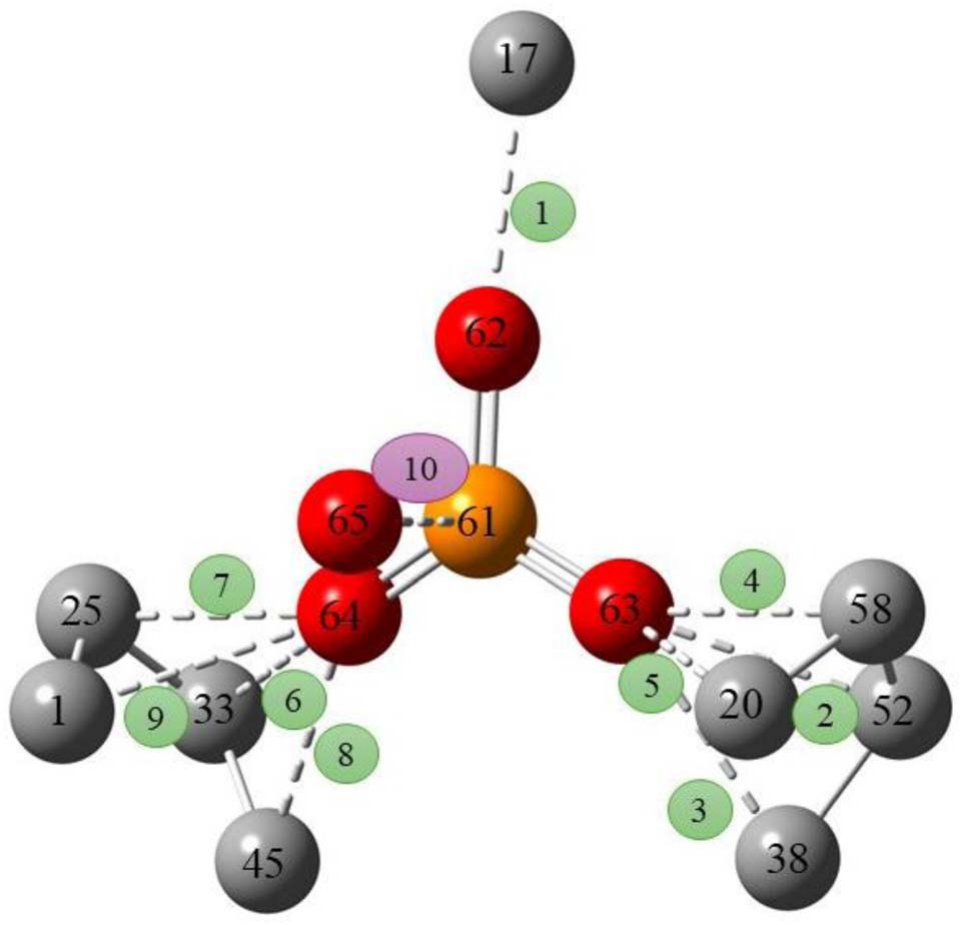
BCPs of the relevant region for QTAIM analysis (green – non-covalent interactions and purple – partial covalent interaction).

The scatter plot of RDG *versus* sign(*λ*2)*ρ* in Fig. 4 corroborates these features, showing that the entire PO_3_ moiety interacts extensively with the C_60_ cage and with the O65 atom covalently bonded to the cage. The internal region of the cage is delineated by alternating red and blue domains, suggesting that a complex combination of repulsive steric contributions and strong, attractive interactions stabilizes the internalized phosphate moiety. For clarity, Fig. 5 provides a simplified representation highlighting only the most relevant regions involved in these contacts. QTAIM analysis further characterizes these interactions through multiple bond critical points (BCPs). A total of 10 BCPs are identified and labelled in Fig. 5, and their corresponding topological parameters are summarized in Table 2.

The topological analysis reveals that for all the critical points, except the tenth, the total electron density (*ρ*) lies between 0.03 and 0.04 au, accompanied by a positive Laplacian of electron density (∇^2^*ρ*), positive total energy density (*H*), and −[*G*/*V*] ratios exceeding 1. In contrast, the 10th critical point, which connects atoms P61 and O65, shows a slightly higher *ρ* value of 0.06 a.u., a positive ∇^2^*ρ*, a negative *H*, and a reduced –[*G*/*V*] ratio of approximately 0.7. Following the criterion of Kumar *et al.*,^86^ non-covalent bonds are identified when the ∇^2^*ρ* is positive, *H* is positive and −*G*/*V* is > 1, with electron density below about 0.03 au. By the same criterion, partially covalent bonds show a higher density of around 0.06 au, ∇^2^ρ Laplacian, negative H, and −G/V below 1. According to this framework, the P61–O65 bond critical point falls into the partially covalent category. In contrast, the C–O bond critical points show a non-covalent nature based on their combined topological features.

This interpretation aligns with the structural evolution during complexation, suggesting atom O65, initially covalently bonded to P61, partially retains this linkage while forming new contacts with the fullerene cage. The tenth BCP thus represents a partial covalent bond.

**Fig. 6:**
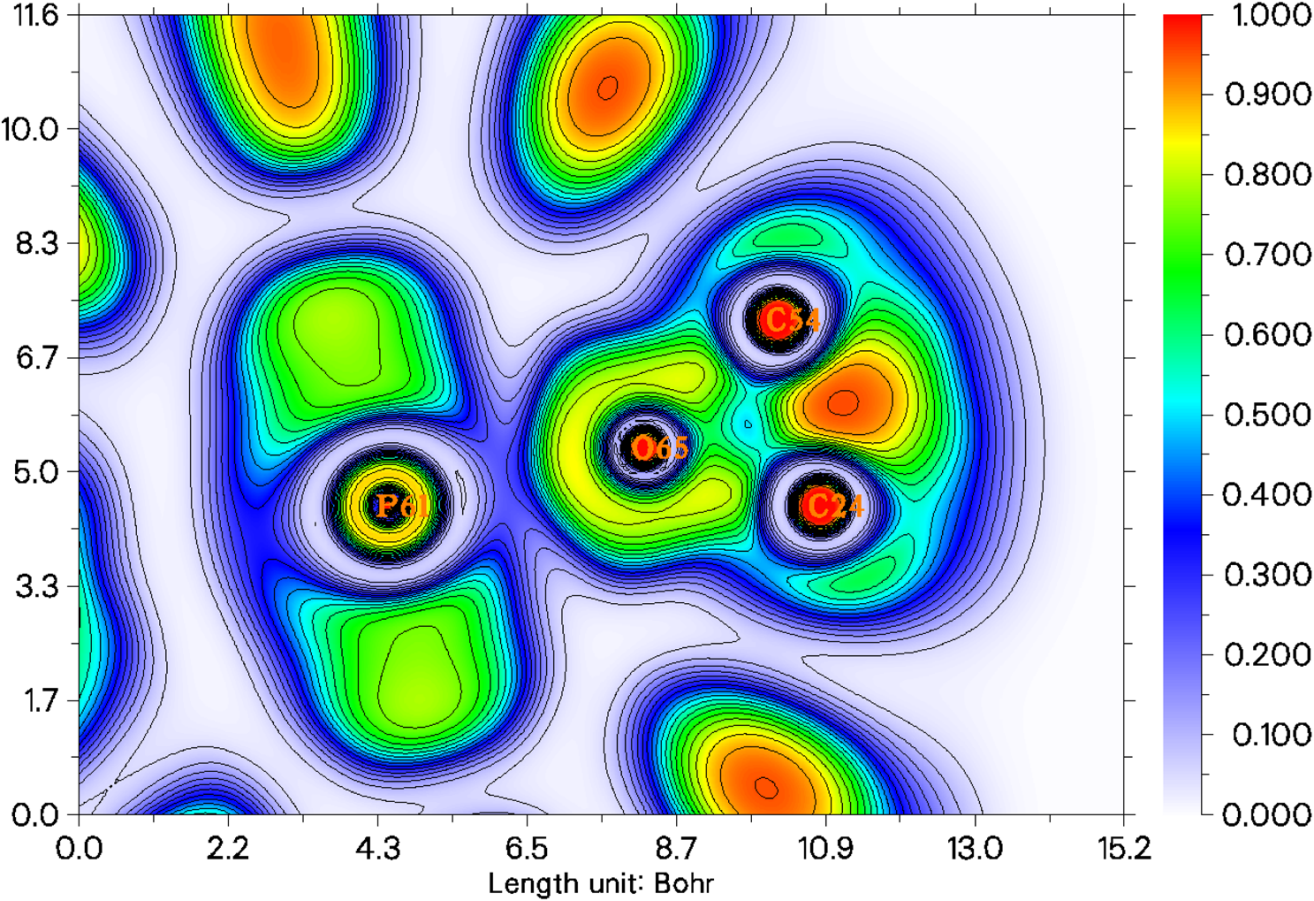
Color-filled 2D ELF contour map of the POC interaction region.

To further elucidate the nature of the chemical bonding within the POC complex, electron localization function (ELF) analysis was performed. ELF provides a robust measure of electron localization, where values near 1.0 indicate highly localized electrons and lower values represent regions of more delocalized or depleted electron density. As illustrated in the color-coded 2D ELF map in Fig. 6, distinct regions of high electron localization are observed between the O65 atom and the cage carbons C54 and C24, clearly delineating the formation of new covalent bonds and confirming the internal oxidation of the fullerene cage. A significant localized basin is also visible between P61 and O65. While the bond has elongated, the sustained localization in this region supports the QTAIM finding of a partial covalent character, indicating that the fragmentation of the phosphate guest is not total but results in a stable, internally bonded hybrid.

### 3.5 NBO analysis

Table 3 summarizes the second-order perturbation theory analysis of the Fock matrix in the NBO basis, highlighting the interactions of P61, O65, C24, and C54 (cage carbons). The analysis reveals an intense delocalization of electron density from the lone pair of the partially fragmented oxygen atom (O65) to the phosphorus center (P61). The LP(2)O65 → LP*(1)P61 interaction exhibits a stabilization energy of 54.65 kcal mol^−1^. This supports the partial covalent character of the P61–O65 bond, ensuring that the partially fragmented oxygen moiety remains associated with the phosphorus atom. Furthermore, the internal oxidation of the fullerene cage is confirmed by the significant hyperconjugative interactions between the new bonds formed by O65 with C54 and C24. Specifically, the BD(1)C24–O65 → BD*(1)C54–O65 interaction and its reciprocal partner, BD(1)C54–O65 → BD*(1)C24–O65, each yielding a stabilization energy of 10.77 kcal mol^−1^, confirm the oxidation of the C_60_ cage by the O65 atom. These results are well aligned with the mechanistic pathway and NCI analysis, confirming the predicted electronic architecture of POC. The complete NBO table showing all interactions between the P61, O65, C24, and C54 is displayed in Supplementary Information Table S3.

**Table 3:** Second-Order Perturbation Theory analysis of the Fock Matrix, highlighting the most significant interactions between P61, O65, and interacting C_60_ cage carbons using DFT at the ωB97X-D/6-31 G(d) level of theory

S. no.	Donor NBO(*i*)	Acceptor NBO (*j*)	*E* ^2^ (kcal mol^−1^)	*E*(*j*)–*E*(*i*)	*F*(*i*,*j*)
1	LP(2)O65	LP*(1)P61	54.65	0.88	0.228
2	BD (1)C24–O65	BD*(1)C54–O65	10.77	1.1	0.098
3	BD (1)C54–O65	BD*(1)C24–O65	10.77	1.1	0.098
4	LP*(1)P61	BD*(1)C24–O65	5.61	0.28	0.059
5	LP*(1)P61	BD*(1)C54–O65	5.61	0.28	0.059

### 3.6 FMO analysis and global reactivity descriptors

**Fig. 7:**
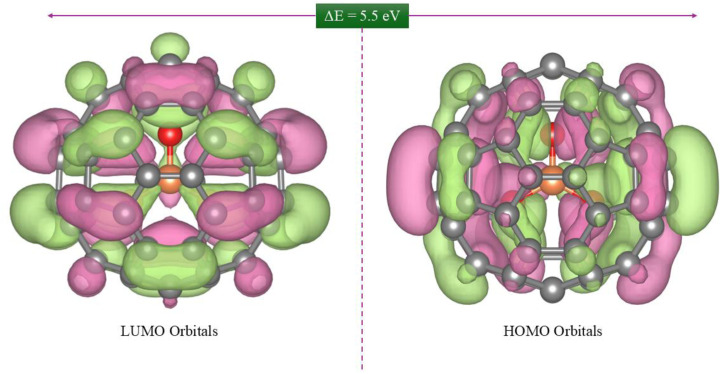
HOMO and LUMO Orbital Structures and Δ*E* of a novel POC, computed using DFT at the ωB97X-D/6-31G(d) level of theory.

The electronic structure of POC was systematically investigated using frontier molecular orbital (FMO) theory,^87^ providing fundamental insights into its chemical reactivity and charge-transfer properties. At the ωB97X-D/6-31 G(d) level, the calculated energies for the highest occupied molecular orbital (HOMO) and the lowest unoccupied molecular orbital (LUMO) were −0.1543 au and 0.0478 au, respectively, yielding a HOMO–LUMO energy gap (Δ*E*) of 0.2021 au, which corresponds to approximately 5.5 eV. As per the results reported by Pearson, E_LUMO_ is defined as the negative of electron affinity, and for many stable molecules, the electron affinity is negative due to intense inter-electronic repulsion resulting in a positive LUMO energy.^88^ The intermediate HOMO–LUMO energy gap is consistently validated by the DFT-CAM-B3LYP/6-31G(d) functional−basis set combination, which predicts a moderate gap of 4.4 eV. The spatial distributions of the HOMO and LUMO for the title complex, along with the HOMO–LUMO energy gap, are displayed in Fig. 7.

Compared with the HOMO–LUMO energy gap of pristine C_60_ of 6.1 eV and 5.0 eV, calculated across the ωB97X-D/6-31G(d) and CAM-B3LYP/6-31G(d) levels of theory, respectively, the POC nanohybrid exhibits a trend of narrowing the HOMO–LUMO energy gap similar to that reported in the literature on endohedral fullerenes.^8,9^ While traditional neutral guests only slightly alter the cage's electronic structure, monoanionic phosphate chemically oxidizes the cage interior and transforms the entire framework into a highly stable, superelectrophilic anion.

Utilizing the conceptual framework of Pearson's Hard–Soft Acid–Base (HSAB) principle,^89^ which links global reactivity descriptors to the polarizability and charge transfer capacities of chemical species, key descriptors such as Ionization potential (I), Electron affinity (A), Electronegativity (*χ*), Chemical hardness (*η*), Electrophilicity index (*ω*), and Global softness (*S*) are calculated using ωB97X-D and successfully cross-validated *via* CAM-B3LYP.^90,91^ The strong agreement between the two mathematical models validates POC as a molecule with intermediate softness and sufficient charge-transfer ability, making it well-suited to participate in chemical reactions in the reaction medium. Table 4 summarizes the comparative frontier molecular orbital analysis between POC and pristine C_60_, and Table 5 displays the global reactivity descriptors for the novel POC.

**Table 4:** The comparative Frontier Molecular Orbitals Analysis between POC and Pristine C60 at the DFT ωB97X-D/6-31 G(d) and CAM-B3LYP/6-31G(d) levels of theory

Parameters	Energy at ωB97X-D/6-31 G(d) level of theory (in au)		Energy at CAM-B3LYP/6-31G(d) level of theory (in au)	
	POC	C60	POC	C60
*E*_LUMO_	0.0478	−0.0656	0.0322	−0.0814
*E*_HOMO_	−0.1543	−0.2879	−0.1302	−0.2636
Δ*E* = *E*_LUMO_ − E_HOMO_	0.2021 au = 5.5 eV	0.2223 au = 6.1 eV	0.1624 au = 4.4 eV	0.1822 au = 5.0 eV

**Table 5:** Global Reactivity Descriptors for the Novel POC Nanohybrid at the DFT ωB97X-D/6-31G(d) and CAM-B3LYP/6-31G(d) levels of theory

S. No.	Global reactivity descriptors	Values (au) at ωB97X-D/6-31G(d)	Values (au) at CAM-B3LYP/6-31G(d)
1	I = −*E*_HOMO_	0.1543	0.1302
2	*A* = −*E*_LUMO_	−0.0478	−0.0322
3	*χ* = (*I* + *A*)/2	0.0533	0.0490
4	*η* = (*I* − *A*)/2	0.1011	0.0896
5	*ω* = *χ*^2^/2*η*	0.0140	0.0133
6	*S* = 1/2*η*	4.9456	5.5804

### 3.7 MESP surface analysis

**Fig. 8:**
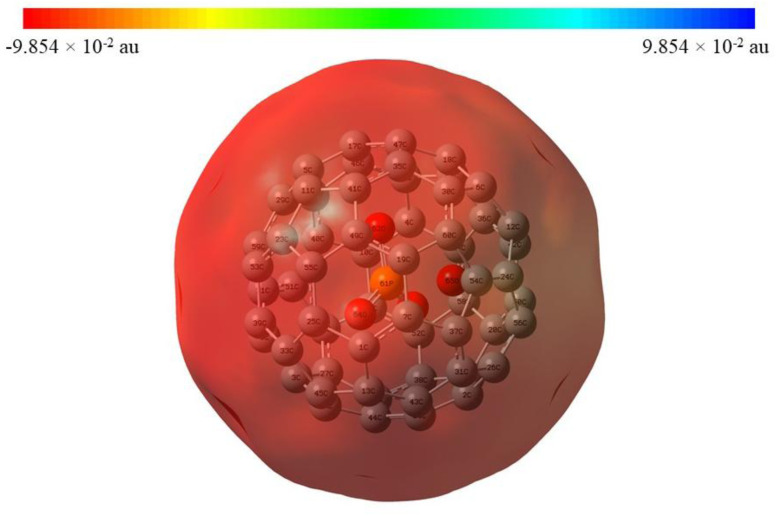
Superelectrophilic MESP surface for Novel POC.

Molecular Electrostatic Potential (MESP) surface identifies electron-rich (red) sites corresponding to electrophilic regions and electron-poor (blue) sites corresponding to nucleophilic regions.^92,93^ Color coding follows the order from red (negative potentials) to green (neutral) to blue (positive potentials). The MESP map of anionic POC shown in Fig. 8 reveals that the internal functionalization of the phosphate unit induces a negative electrostatic environment that extends over the fullerene cage, indicating that the entire POC behaves as a single, delocalized anion. Hence, the phosphate incorporation leads to an MESP surface dominated by a deep red surface covering almost the whole molecular envelope with the most negative values around 9.85 × 10^−2^ au distributed over the carbon cage. No blue regions appear on the outer surface, confirming that the complex exhibits superelectrophilic behaviour. For drug delivery or biomolecular recognition, the observed MESP profile indicates that the POC can serve as a strongly charged nanocarrier in which the electron-rich surface can engage in electrostatic and π–cation interactions with positively charged residues at protein binding sites. At the same time, the endohedral phosphate moiety remains shielded by the carbon cage, so direct covalent reactivity of PO_4_ is suppressed while its electronic influence is transmitted through the functionalized electrostatic landscape of the fullerene shell. Overall, the MESP analysis confirms that endohedral derivatization synergistically transforms the POC framework into a stable superelectrophilic anion, exhibiting promising potential for selective interaction with cationic biological domains.

### 3.8 Comparative molecular docking analysis

**Fig. 9:**
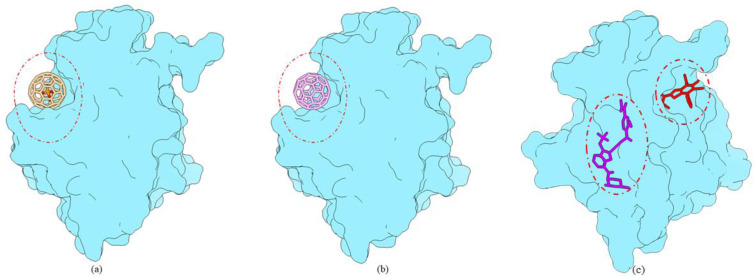
Docked structures of (a) POC (cream), (b) Pristine C_60_ (pink), and (c) Rezatapopt (purple) and MB710 (red) against 5O1G (blue).

**Fig. 10:**
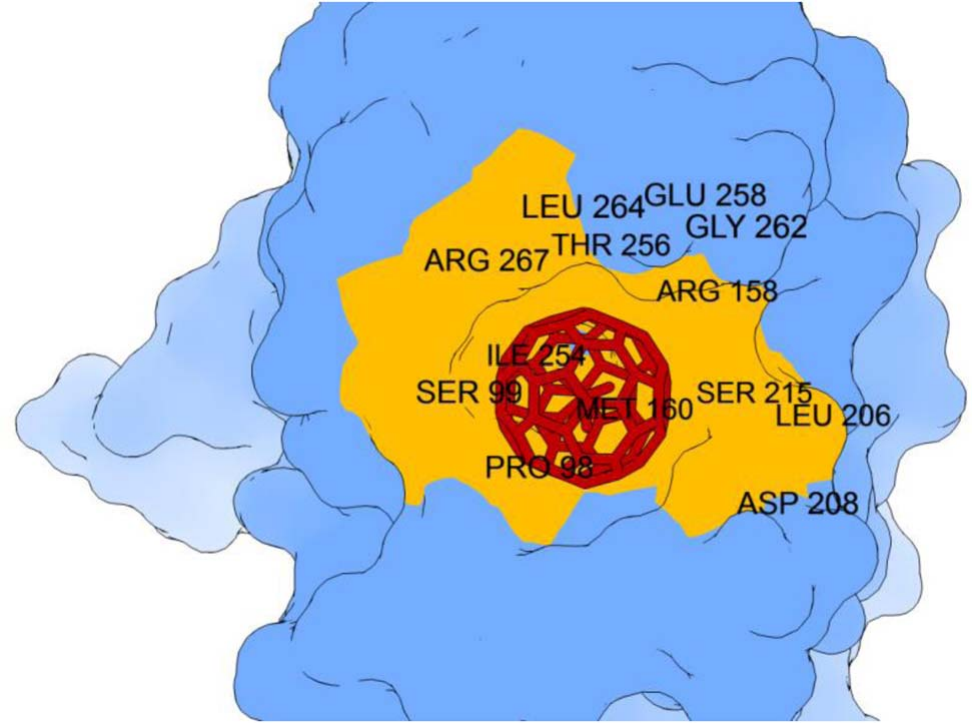
Binding Pocket with labelled residues incorporating POC within the active site.

A comparative insight into the interactions of the POC and pristine C_60_ with the Y220 mutation of the p53 tumour suppressor protein (PDB ID: 5O1G) was obtained through molecular docking using AutoDock Vina. POC shows a binding affinity of −11.5 kcal mol^−1^, which, compared with pristine C_60_ (−10.5 kcal mol^−1^), is significantly higher. These results are benchmarked against MB710 and Rezatapopt, which are documented stabilizers of the Y220C mutation^34,43^ and yield affinities of −5.8 kcal mol^−1^ and −6.1 kcal mol^−1^, respectively, shown in Table 6. The significantly higher binding affinity of POC suggests its potential as a promising stabilizing candidate against the p53–Y220C mutant. Fig. 9 shows the binding poses of POC, C_60_, Rezatapopt, and MB710 against the protein 5O1G. Fig. 10 depicts the binding pocket with labeled residues, showing the novel POC nestled within the active site of the protein surrounded by residues Pro 98, Ser 99, Arg 158, Met 160, Leu 206, Asp 208, Ser 215, Ile 254, Thr 256, Glu 258, Gly 262, Leu 264, and Arg 267, which delineate the binding cavity.

**Table 6:** Comparative molecular docking scores of the ligands against the protein 5O1G

S. No.	Ligand	Binding affinity
1	POC	−11.5 kcal mol^−1^
2	C_60_	−10.5 kcal mol^−1^
3	Rezatapopt	−6.1 kcal mol^−1^
4	MB710	−5.8 kcal mol^−1^

### 3.9 Molecular dynamics analysis

#### 3.9.1 Root mean square deviation (RMSD)

**Fig. 11:**
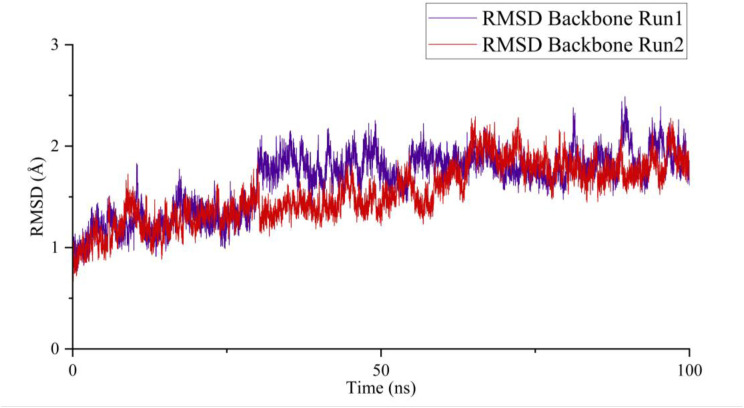
RMSD of protein backbone least squares fit to itself.

**Fig. 12:**
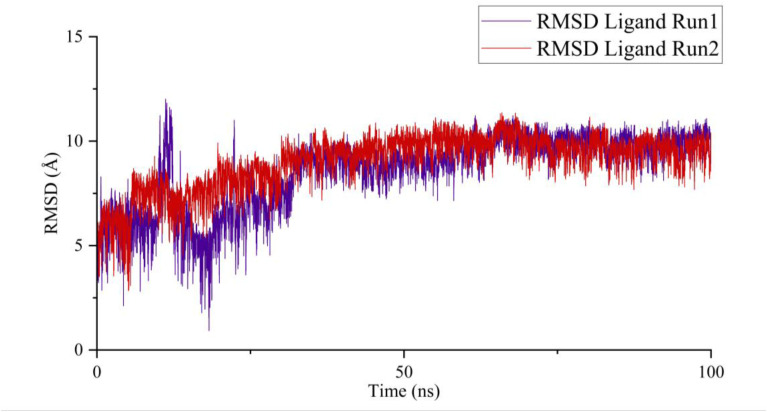
RMSD of ligand least squares fit to protein backbone.

**Fig. 13:**
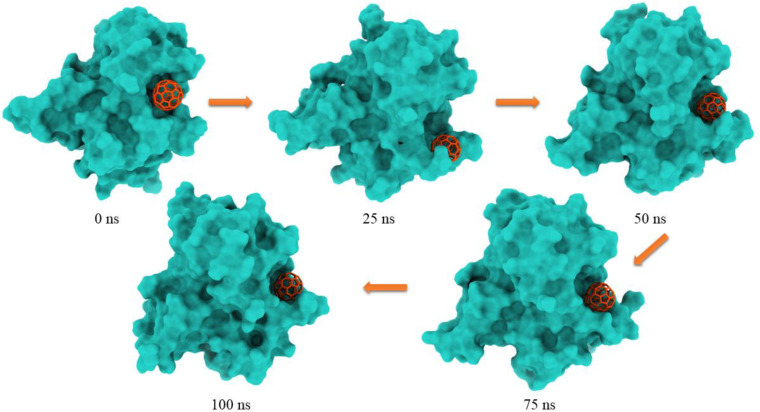
Time-series snapshots of the 100 ns simulation for the protein (blue)–ligand (orange) complex during MD run 2.

Root Mean Square Deviation (RMSD) analyses were performed for both the protein and ligand across two simulation runs. RMSD calculates the average distance produced by the displacement of specific atoms over a certain period from a reference frame.^94^ As depicted in Fig. 11 and 12, run 2 demonstrates higher stability than run 1, indicating that the extended equilibration period applied in run 2 resulted in a more stable molecular dynamics trajectory. Both runs produced comparable outcomes, each reaching stable RMSD plateaus within the 100 ns MD trajectory. The protein backbone RMSD, fitted by least squares, stabilized at approximately 30 ns in run 1 and around 65 ns in run 2. The ligand RMSD, aligned to the protein backbone, exhibited consistent stability at approximately 35 ns in both runs. Gradual increases in the protein backbone RMSD, fluctuating between 0.75 Å and 2.5 Å throughout the simulation, are indicative of conformational adjustments in the protein structure driven by the presence of POC. Notably, the ligand RMSD profile exhibited a shift, reaching a peak value of approximately 11 Å within the initial 50 ns. This deviation corresponds to the spherical cage undergoing localized rotational spinning and translational sliding within the spacious Y220C oncogenic cavity as it transitions to a more favorable, MD-equilibrated pose, as evident from Fig. 13. Importantly, the ligand remains continuously confined within the Y220C cavity throughout the trajectory.

**Fig. 14:**
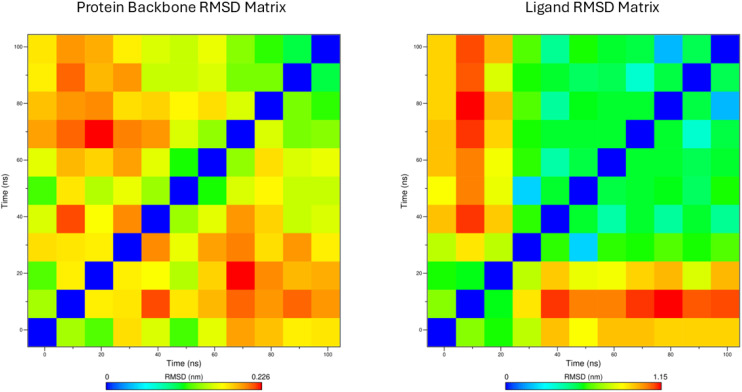
Pairwise 2D-RMSD matrices for protein backbone and ligand for run 2 over the 100 ns simulation time.

From 50 ns onward, no prominent changes are observed in the RMSD profiles of the ligand and protein backbone, suggesting that the ligand attains a stable position within the protein cavity, as evident from Fig. 13. The achievement of this stable plateau after the 11 Å shift confirms that the system has reached a localized energy minimum. Crucially, the absence of abrupt RMSD fluctuations or sudden jumps throughout the simulation confirms robust system stability across both trajectories. Since run 2 is considered more stable, the RMSD matrix heatmaps, which display the pairwise structural deviations over a 100 ns simulation time for the protein backbone and the ligand, are shown in Fig. 14.

#### 3.9.2 Root mean square fluctuation (RMSF)

**Fig. 15:**
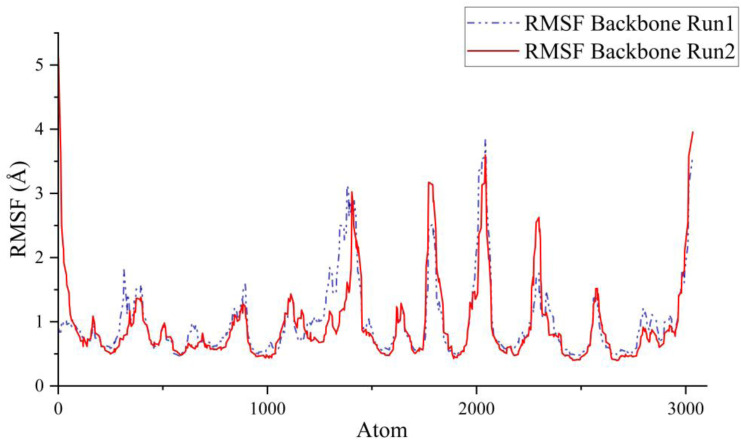
RMS Fluctuations for protein backbone.

**Fig. 16:**
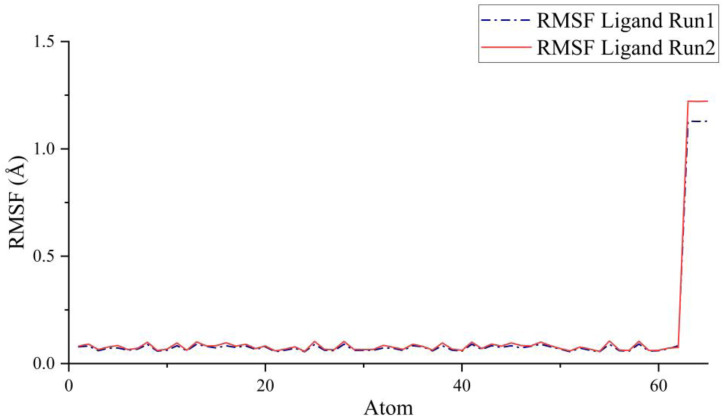
RMS Fluctuations for ligand (POC).

The root mean square fluctuations (RMSF) for both molecular dynamics runs were compared to assess the atomic flexibility of the protein backbone and ligand.^95^ As shown in Fig. 15 and 16, the RMSF profiles of both runs exhibit similar trends for the protein backbone and the bound ligand. Up to the 103rd atom, run 2 displays slightly higher RMSF values than run 1, likely due to its longer equilibration period, making these values more representative of the equilibrated system. The overall RMSF landscape reveals several flexible regions, with a maximum fluctuation amplitude of approximately 5.1 Å. In contrast, POC demonstrates remarkable structural stability, with fluctuations below 0.1 Å for the cage framework. The phosphorus and oxygen atoms exhibit moderate mobility, with RMSF values around 1.2 Å. These fluctuations are marginally higher in run 2, again attributed to extended equilibration, and are thus considered more reliable.

#### 3.9.3 PCA analysis

**Fig. 17:**
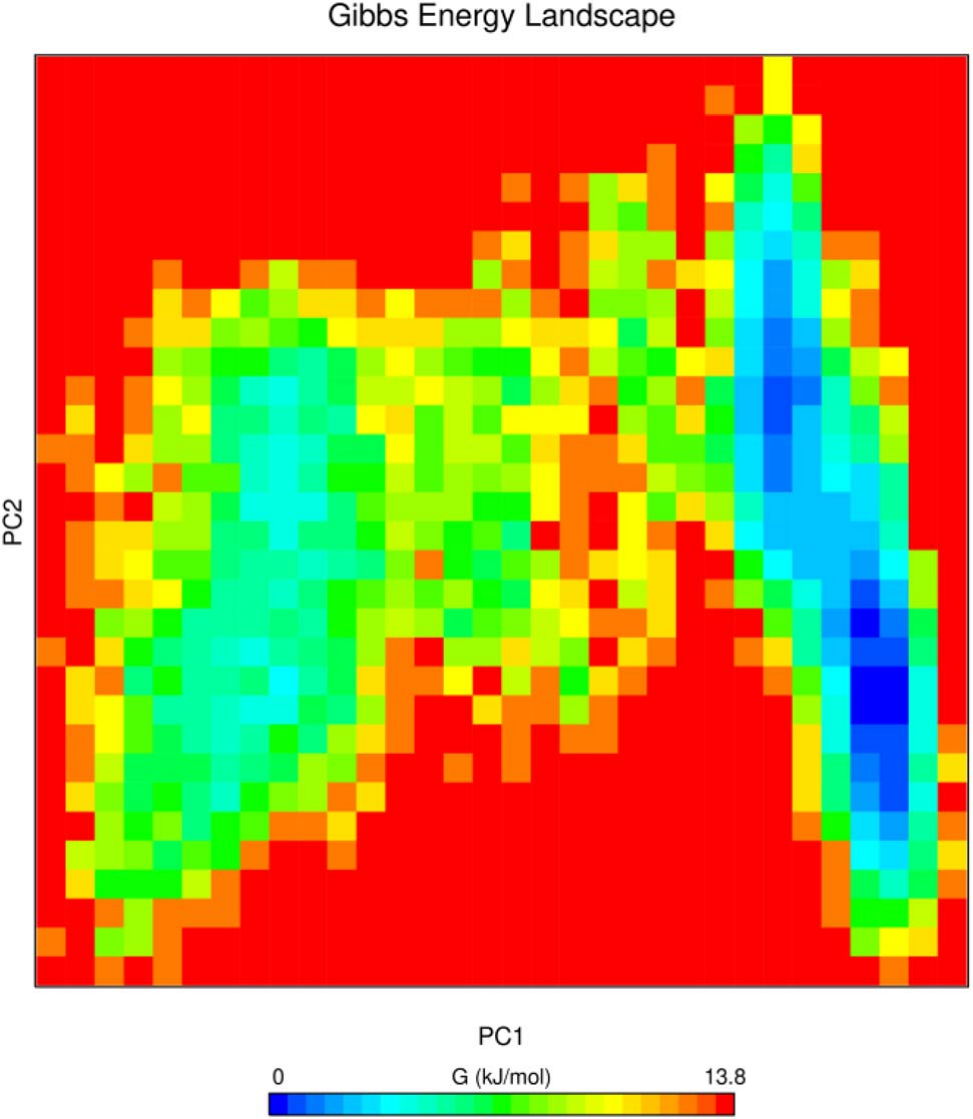
Gibbs Free Energy Landscape for protein–ligand complex.

The Gibbs free energy landscape (FEL) analysis shown in Fig. 17, focused on the protein backbone–ligand complex, provides a vivid and integrated depiction of its conformational dynamics and stability over the course of the molecular dynamics simulation. By projecting the trajectory onto the principal components PC1 and PC2, the FEL reveals distinct energetic basins, where deep blue zones mark the most stable, low-energy conformational states, and vibrant red regions denote energetically unfavorable configurations. Areas shaded in cyan and green illustrate intermediate, semi-stable states that the complex explores during simulation, reflecting its inherent flexibility.

Towards the left half of the Gibbs Energy Landscape, a large area of green and cyan hue is observed, reflecting semi-stable states during the simulation. Notably, two deep blue regions stand out on the right side of the landscape, encircled by lighter blue hues. These regions highlight the predominant, stable conformations the protein backbone–ligand complex consistently adopts during the trajectory, indicating a stable backbone with a well-defined binding pose of the ligand. The sustained occupation of low-energy states throughout the FEL landscape highlights the structural integrity and stability of the protein backbone–ligand complex throughout the trajectory.

#### 3.9.4 SASA of free and bound ligand along with burial percentage of POC

**Fig. 18:**
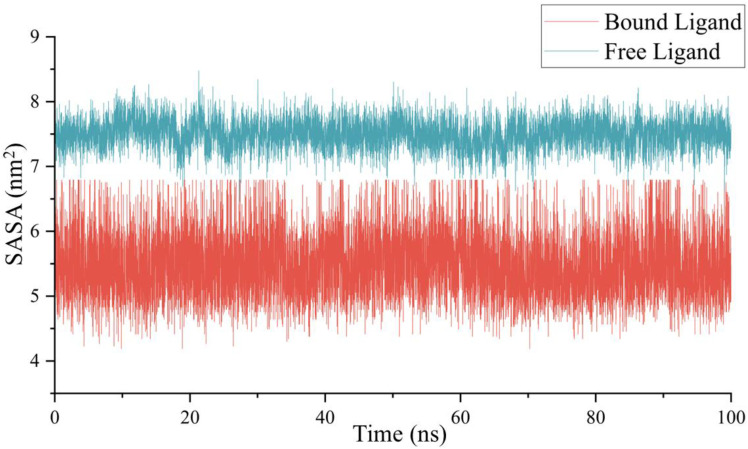
SASA for free and bound ligand for run 2.

The solvent accessible surface area (SASA) graph for the free and bound ligand has been evaluated and displayed in Fig. 18. The SASA remains stable, suggesting persistence of the ligand within the binding pocket of the 5O1G cavity. Burial percentage can be calculated by using eqn (10):10
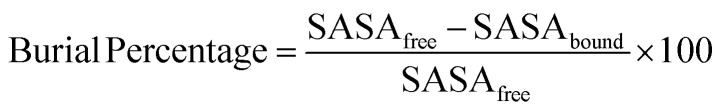


The average SASA for the free ligand (SASA_free_) is 7.50 nm^2^, while for the bound ligand (SASA_bound_) it is 5.48 nm^2^. Substituting these values into eqn (10), we calculate the burial percentage of POC to be 26.9% within the cavity of the p53 Y220C mutant.

### 3.10 Energy calculations

The MM/PBSA and MM/GBSA analyses, which study the binding of the novel POC with the p53 Y220C mutated protein, reveal several important energy components contributing to the thermodynamically favorable binding shown in Table 7. Energy calculations have been performed over the final 30 ns of MD run 2, as the RMSD analysis indicated good structural stability of both the ligand and the protein backbone beyond 70 ns. The binding free energy (Δ*G*_bind_) is predicted as −38.78 ± 3.46 kcal mol^−1^ and −43.73 ± 3.06 kcal mol^−1^ for PB and GB models, respectively, indicating strong and stable binding across both models.

**Table 7:** Interaction energy terms for the protein–ligand complex

Energy components	MMPBSA		MMGBSA	
	Average (kcal mol^−1^)	Standard deviation	Average (kcal mol^−1^)	Standard deviation
ΔVDWAALS	−57.11	3.34	−57.11	3.34
ΔEEL	−0.72	1.07	−0.72	1.07
ΔEPB	15.06	2.19	—	—
ΔEGB	—	—	9.16	2.15
ΔESURF	—	—	−2.33	0.19
ΔENPOLAR	−3.18	0.12	—	—
ΔEDISPER	0.00	0.00	—	—

**Summary**				
ΔGGAS	−57.83	3.57	−57.83	3.57
ΔGSOLV	11.88	2.16	6.83	2.20
TΔS	7.17	0.05	7.27	0.05
Δ*G*_bind_	−38.78	3.46	−43.73	3.06

Key contributions from the molecular mechanics terms indicate that van der Waals interactions (ΔVDWAALS) are the dominant favorable forces, with an energy of −57.11 ± 3.34 kcal mol^−1^ across both models. Electrostatic interactions (ΔEEL) contribute minimally at −0.72 ± 1.07 kcal mol^−1^ across both models, indicating a minor role in the overall binding affinity relative to van der Waals forces. The solvation energy components partially offset the favorable gas–phase interactions for both models. The overall gas–phase energy (ΔGGAS) sums to −57.83 ± 3.57 kcal mol^−1^ for both models, and when combined with solvation energies (ΔGSOLV) of +11.88 ± 2.16 kcal mol^−1^ and +6.83 ± 2.16 kcal mol^−1^ and entropy terms (*T*Δ*S*) of 7.17 ± 0.05 kcal mol^−1^ and 7.27 ± 0.05 kcal mol^−1^ for PB and GB models respectively, results in the net binding free energy reported. Although ΔGGAS and *T*Δ*S* exhibit comparable values in both PB and GB models, the variation in ΔGSOLV primarily accounts for the observed differences in Δ*G*_bind_. Since the MM/PBSA model provides a more rigorous treatment of polar solvation energy, the binding energy of −38.78 ± 3.46 kcal mol^−1^ obtained from this approach is considered to offer a more reliable estimation for the system.

### 3.11 Toxicity and safety profile

The toxicity and safety profile of the complex reveal a topological polar surface area (TPSA) of 93.53 Å^2^ with a molecular weight of 818.64 g mol^−1^. The acute oral toxicity (LD_50_) of the POC nanohybrid is estimated to be 650 mg kg^−1^, classifying it within Toxicity Class 4. This classification indicates a moderate acute toxicity profile, falling well within acceptable parameters for carefully dosed preclinical evaluations. Predictive screening for specific organ and systemic toxicities revealed a favorable safety profile. The nanohybrid is predicted to be inactive for hepatotoxicity, neurotoxicity, nephrotoxicity, and cardiotoxicity. Furthermore, it demonstrates inactivity across severe endpoints, including carcinogenicity, immunotoxicity, and overall cytotoxicity. The complex also exhibits an inert metabolic profile, with predicted inactivity against all major cytochrome P450 enzyme isoforms.

Despite the broadly favorable systemic profile, the computational model did flag the nanohybrid as active for mutagenicity (probability 0.66) and respiratory toxicity (probability 0.55). At the molecular level, specific Molecular Initiating Events are highlighted, showing probable binding and activity for Transthyretin with a probability of 0.69 and the Pregnane X Receptor with a probability of 0.57. Moreover, the complex is predicted to be active for blood–brain barrier (BBB) permeation (probability 0.78). While BBB permeability can be highly advantageous for targeting central nervous system malignancies or brain metastases, the predicted mutagenic and respiratory endpoints highlight the importance of localized administration. The complete table of the *in silico* toxicity report is displayed in Supplementary Information Table S4. Conclusively, these computational predictions characterize the POC nanohybrid as a preliminary candidate for a pharmacological chaperone, with subsequent *in vitro* and *in vivo* validations to refine targeted delivery and mitigate off-target risks.

## 4 Conclusion

The encapsulation of phosphate within the C60 cage induces a distinct structural rearrangement which extends beyond traditional host–guest interactions. Unlike conventional endofullerenes, characterized by the passive encapsulation of neutral molecules *via* non-covalent forces, the designed POC nanohybrid undergoes a spontaneous molecular metamorphosis. The oxygen atom (O65) partially dissociates from its original covalent bond with the phosphorus atom (P61) and forms new covalent bonds with two carbon atoms of the fullerene cage. Non-covalent interaction (NCI) analysis reveals a balance of attractive and repulsive forces that stabilize the embedded PO_3_ moiety within the cage, with QTAIM analysis indicating that O65 retains partial covalent character with P61 validated *via* NBO analysis. The permanence of this structural rearrangement is mathematically corroborated by a large recovery time of approximately 10^87^ years, suggesting the complex remains thermodynamically stable and highly persistent under ambient conditions. FMO analysis identifies a moderate HOMO–LUMO gap of 5.5 eV, indicative of good chemical stability coupled with suitable reactivity descriptors. MESP mapping further characterizes POC as a superelectrophilic anion, promoting strong interactions with cationic and polar residues in protein environments.

Molecular docking and extended molecular dynamics simulations demonstrate high binding affinity and stable interaction of POC with the p53 Y220C (PDB ID: 5O1G) mutant, exhibiting improved binding affinity of −11.5 kcal mol^−1^ compared to pristine C_60_ (−10.5 kcal mol^−1^), along with MB710 and Rezatapopt with binding affinities of −6.1 and −5.8 kcal mol^−1^, respectively. The 5O1G-POC complex maintains structural stability over 100 nanosecond trajectories, suggesting that POC effectively stabilizes the mutant tumor suppressor conformation. The binding free energies of −38.78 ± 3.46 kcal mol^−1^ and −43.73 ± 3.06 kcal mol^−1^ for the PB and GB models suggest strong engagement of POC with 5O1G, with a burial percentage of 26.9%, providing a promising alternative approach that directly stabilizes the immunogenic Y220C mutant. The functionality of this nanostructure with protein-stabilizing properties positions POC as a novel and promising candidate for targeted p53 cancer mutant Y220C therapeutics. Furthermore, predictive toxicological screening positions POC as a viable preclinical candidate with a moderate acute toxicity profile of Class 4. While the model indicates advantageous blood–brain barrier (BBB) permeability, flagged risks for mutagenicity and respiratory toxicity underscore the necessity for localized administration strategies.

While current pharmacological chaperones, like Rezatapopt, for the p53–Y220C reactivation often face constraints such as susceptibility to resistance and binding affinity ceilings,^96^ the POC presents a structurally distinct candidate with promising high-affinity binding characteristics. The internally functionalized, superelectrophilic architecture of this POC nanohybrid represents a novel structural motif within fullerene chemistry. However, as these promising insights are purely *in silico*, translating this computational roadmap into tangible therapeutic strategies requires experimental validation. Future research is invited to prioritize the physical synthesis and isolation of the POC nanostructure, followed by *in vitro* and *in vivo* evaluations of p53 restoration in specific Y220C mutant cell lines. Ultimately, the metamorphosed POC nanohybrid illuminated in this study provides a valuable foundational framework for the future development of targeted cancer therapeutics.

## Ethical approval

The authors declare that they have adhered to the journal's ethical guidelines and that this study was conducted with the utmost respect for the rights and dignity of all participants.

## Author contributions

Conceptualization, A. K. M.; validation, R. J. L. and A. K. M.; investigation, R. J. L., S. K. S., and A. K. M; data curation, R. J. L. and A. K. M.; writing—original draft preparation, R. J. L., S. K. S., and A. K. M.; writing—review and editing, A. K. M.; visualization, R. J. L., S. K. S., and A. K. M; supervision, A. K. M. All authors have read and agreed to the published version of the manuscript.

## Conflicts of interest

The authors report no conflicts of interest or competing interests.

## Supplementary Material

RA-OLF-D6RA01727D-s001

## Data Availability

All relevant data analysed during this study are included in this published article. Supplementary information is available. See DOI: https://doi.org/10.1039/d6ra01727d.
