# Supplementary material for: DFT design of PO4@C60 nanohybrid unveils superelectrophilic molecular metamorphosis with molecular dynamics exhibiting therapeutic potential against p53 cancer mutant Y220C
Source: RSC Adv. 2026 Jul 2;16(34):32744–62. doi: 10.1039/d6ra01727d (PMC13325430; doi:10.1039/d6ra01727d)
Supplement: RA-016-D6RA01727D-s001 [file RA-016-D6RA01727D-s001.pdf]

Supplementary Information (SI) for RSC Advances.
This journal is © The Royal Society of Chemistry 2026

Supplementary Information

Title: DFT Design of PO4@C60 Nanohybrid unveils Superelectrophilic Molecular Metamorphosis with Molecular Dynamics exhibiting its Therapeutic Potential against p53 Cancer Mutant Y220C

Ryan John Laverne[1], Sudheesh K. Shukla[2,3*], Ashok Kumar Mishra[1*]

[1]Department of Physics, Dr. Shakuntala Misra National Rehabilitation University, Lucknow, Uttar Pradesh-226017, India.
[2]Department of Biosciences, Graphic Era (Deemed to be University), Dehradun, Uttarakhand-248002, India.
[3]Department of Chemical Sciences, University of Johannesburg, Doornfontein Campus, P.O. Box 17011, Johannesburg 2028, South Africa.

[*]Corresponding Author E-mail: akmishra@dsmnru.ac.in, akmishra2k5@gmail.com (AKM).

Table S1. Structural Parameters for Novel POC calculated using DFT at the ωB97X−D/6−31G(d) level of theory.

Atom (with numbering)	Bond Atom (with numbering)	Bond Length (Å)	Angle Atom (with numbering)	Angle (°)	2nd Angle Atom (with numbering)	2nd Angle (°)	2nd Angle Type
C(7)	C(1)	1.405					
C(13)	C(1)	1.459	C(7)	118.835			
C(25)	C(1)	1.477	C(7)	118.948	C(13)	108.882	Pro-R
C(19)	C(7)	1.495	C(1)	120.664	C(13)	136.695	Dihedral
C(37)	C(7)	1.440	C(1)	119.045	C(19)	109.154	Pro-R
C(31)	C(37)	1.393	C(7)	123.194	C(1)	5.879	Dihedral
C(54)	C(37)	1.496	C(7)	102.248	C(31)	114.026	Pro-S
C(24)	C(54)	1.470	C(37)	122.007	C(7)	-145.573	Dihedral
C(60)	C(54)	1.493	C(24)	121.463	C(37)	113.769	Pro-R
C(30)	C(60)	1.397	C(19)	123.589	C(54)	115.001	Pro-S
C(49)	C(19)	1.389	C(7)	119.761	C(60)	117.276	Pro-S
O(65)	C(54)	1.429	C(24)	59.047	C(37)	98.311	Pro-R
C(12)	C(24)	1.492	C(54)	121.515	O(65)	98.258	Pro-S
C(56)	C(24)	1.496	C(12)	113.770	C(54)	121.955	Pro-S
C(6)	C(30)	1.520	C(60)	122.676	C(19)	-133.920	Dihedral
C(18)	C(6)	1.441	C(12)	118.991	C(30)	107.190	Pro-R
C(35)	C(30)	1.441	C(6)	107.190	C(60)	119.010	Pro-R
C(42)	C(12)	1.443	C(6)	123.574	C(24)	100.559	Pro-S
C(26)	C(56)	1.393	C(24)	114.053	C(12)	-151.074	Dihedral
C(2)	C(26)	1.434	C(31)	107.870	C(56)	117.621	Pro-R
C(43)	C(31)	1.434	C(26)	107.870	C(37)	117.627	Pro-S
C(14)	C(43)	1.467	C(13)	121.530	C(31)	106.753	Pro-R
C(8)	C(2)	1.419	C(14)	121.530	C(26)	120.901	Pro-R
C(44)	C(14)	1.376	C(2)	119.473	C(43)	119.455	Pro-S
C(45)	C(13)	1.468	C(1)	107.738	C(43)	118.730	Pro-R
C(50)	C(56)	1.441	C(24)	102.178	C(26)	123.230	Pro-S
C(20)	C(50)	1.405	C(42)	120.664	C(56)	119.012	Pro-S
C(36)	C(42)	1.389	C(12)	117.337	C(50)	119.726	Pro-S
C(38)	C(8)	1.468	C(2)	118.699	C(20)	107.771	Pro-R
C(58)	C(20)	1.477	C(8)	108.882	C(50)	118.948	Pro-R
C(15)	C(44)	1.466	C(14)	119.621	C(2)	-141.810	Dihedral
C(3)	C(15)	1.445	C(44)	105.724	C(45)	120.289	Pro-S
C(32)	C(44)	1.466	C(14)	119.621	C(15)	110.477	Pro-S
C(27)	C(32)	1.445	C(38)	120.352	C(44)	105.686	Pro-S

C(9)	C(3)	1.388	C(15)	119.883	C(27)	120.340	Pro-R
C(33)	C(45)	1.476	C(13)	108.124	C(15)	119.903	Pro-R
C(39)	C(33)	1.400	C(25)	118.169	C(45)	118.838	Pro-R
C(21)	C(9)	1.456	C(3)	118.999	C(39)	108.748	Pro-S
C(52)	C(38)	1.476	C(8)	108.089	C(32)	119.839	Pro-S
C(22)	C(52)	1.399	C(38)	118.878	C(58)	118.205	Pro-R
C(28)	C(58)	1.403	C(20)	119.428	C(52)	119.720	Pro-S
C(4)	C(28)	1.454	C(36)	105.171	C(58)	121.519	Pro-S
C(48)	C(36)	1.457	C(28)	110.244	C(42)	120.046	Pro-R
C(16)	C(48)	1.450	C(18)	119.626	C(36)	107.392	Pro-S
C(10)	C(4)	1.383	C(16)	120.194	C(28)	118.661	Pro-S
C(40)	C(10)	1.453	C(4)	118.324	C(22)	109.625	Pro-S
C(46)	C(16)	1.399	C(4)	121.764	C(48)	119.832	Pro-R
C(47)	C(18)	1.477	C(6)	109.364	C(48)	120.977	Pro-S
C(17)	C(47)	1.401	C(18)	119.312	C(35)	119.312	Pro-R
C(5)	C(17)	1.481	C(46)	106.819	C(47)	119.016	Pro-R
C(34)	C(46)	1.456	C(16)	117.490	C(17)	108.036	Pro-R
C(29)	C(34)	1.454	C(40)	119.384	C(46)	108.536	Pro-R
C(11)	C(5)	1.399	C(17)	121.236	C(29)	117.461	Pro-S
C(41)	C(35)	1.410	C(30)	117.975	C(47)	120.977	Pro-S
C(23)	C(11)	1.468	C(5)	121.767	C(41)	106.990	Pro-R
C(51)	C(40)	1.443	C(10)	106.892	C(34)	120.684	Pro-R
C(53)	C(39)	1.462	C(9)	106.149	C(33)	121.189	Pro-S
C(55)	C(25)	1.404	C(1)	119.430	C(33)	119.729	Pro-R
C(57)	C(27)	1.388	C(3)	120.340	C(32)	119.853	Pro-R
C(59)	C(21)	1.444	C(9)	108.582	C(51)	119.911	Pro-R
O(62)	C(59)	3.171	C(21)	96.229	C(29)	57.081	Pro-R
P(61)	O(62)	1.437	C(1)	47.622	C(7)	108.142	Dihedral
O(63)	P(61)	1.437	O(62)	119.235	C(1)	-167.962	Dihedral
O(64)	P(61)	1.437	O(62)	119.235	O(63)	120.213	Pro-R

where, Pro-R and Pro-S are stereochemical descriptors.

Table S2. Calculated Frequencies and corresponding IR Intensities using DFT at the ωB97X−D/6−31G(d) level of theory.

**Mode**	**Frequency**	**IR Intensity**
1.	7.5501	0.0099
2.	129.4441	0.1028
3.	163.8911	0.2989
4.	226.3650	0.2310
5.	245.7638	0.5892
6.	266.2866	0.4676
7.	272.6490	1.4122
8.	301.3577	0.7597
9.	301.9664	0.1967
10.	308.1280	2.8748
11.	312.6007	3.2236
12.	344.5271	3.5321
13.	349.4546	0.3063
14.	356.4373	2.4762
15.	373.2552	3.3345
16.	387.8923	0.4240
17.	400.4244	0.0697
18.	401.7188	0.0367
19.	402.0898	0.0036
20.	402.7453	0.0538
21.	405.4572	0.0495
22.	405.4902	0.0047
23.	410.6572	0.0020
24.	430.0540	0.4537
25.	447.1692	0.2884
26.	450.5449	0.7864
27.	464.0284	0.8258
28.	470.7298	0.1671
29.	471.6769	0.6528
30.	491.8757	0.3207
31.	502.8737	0.4889
32.	512.0426	3.1788
33.	520.1845	2.7126
34.	522.2772	6.0986
35.	526.2780	8.6009
36.	526.9922	3.0313
37.	540.8321	25.5027
38.	543.5802	6.9359
39.	545.8447	9.8422
40.	555.5835	13.4307
41.	556.2175	1.6486
42.	557.5207	1.5333
43.	560.0378	19.1014
44.	565.1271	6.4721
45.	565.5113	1.4818
46.	565.7304	0.2074
47.	567.4628	0.0027
48.	569.9701	0.2052

49.	570.8451	7.5167
50.	571.4038	0.0051
51.	578.4203	1.7264
52.	579.4723	0.7234
53.	581.2165	0.2170
54.	583.1395	1.6213
55.	587.7720	7.4339
56.	608.1761	21.9259
57.	613.0491	14.7853
58.	639.5624	18.1713
59.	660.6673	2.4786
60.	666.3721	0.6449
61.	672.2107	8.4970
62.	672.6331	3.0748
63.	679.4110	0.5528
64.	680.9881	0.0134
65.	683.9035	0.7307
66.	687.0800	6.0560
67.	688.0235	3.8637
68.	694.3226	1.3339
69.	702.2329	12.8998
70.	703.5357	3.0065
71.	713.2541	0.0001
72.	715.5791	0.5662
73.	721.6391	2.8713
74.	723.1186	1.2398
75.	726.3676	3.2674
76.	728.2489	4.1884
77.	729.4078	0.0477
78.	733.8223	5.3658
79.	735.9175	0.1007
80.	736.8221	0.7310
81.	738.4507	0.0277
82.	739.8519	0.7708
83.	739.9749	1.8881
84.	743.1032	0.0409
85.	746.6839	1.2861
86.	748.5433	0.0127
87.	753.0836	0.0131
88.	754.0524	4.8211
89.	757.3856	0.2484
90.	760.9908	0.0262
91.	762.0058	5.6030
92.	764.0696	2.3000
93.	777.0229	0.2681
94.	781.3833	9.2751
95.	783.7350	1.9997
96.	786.9177	0.6439
97.	793.7297	0.0217
98.	799.3129	0.0001
99.	802.0989	0.1628
100.	835.0146	0.0182
101.	836.7198	0.1489

102.	840.0682	0.0111
103.	900.8805	3.3896
104.	925.5729	2.8634
105.	936.2981	0.1201
106.	938.0562	0.3340
107.	943.7418	4.8306
108.	964.1310	0.0002
109.	968.9021	4.3664
110.	969.7751	0.3375
111.	978.2288	0.0021
112.	1016.7629	8.6039
113.	1029.6639	0.1150
114.	1035.1036	0.0553
115.	1041.5795	0.7731
116.	1079.1787	4.7277
117.	1085.2129	2.0630
118.	1105.2321	2.8372
119.	1110.3942	0.6315
120.	1113.7999	3.1236
121.	1117.3448	0.0820
122.	1123.9026	0.2778
123.	1141.7599	0.4343
124.	1169.7781	6.6525
125.	1172.2145	5.7702
126.	1187.1589	1.2406
127.	1207.4563	3.5500
128.	1212.9232	2.2240
129.	1221.5400	5.2029
130.	1226.9414	0.1759
131.	1235.1250	1.0519
132.	1247.0585	0.5569
133.	1250.7780	1.0107
134.	1255.4353	0.7775
135.	1261.2195	0.2930
136.	1262.4887	0.2999
137.	1269.3978	0.5165
138.	1270.6385	0.2950
139.	1279.7480	1.0993
140.	1283.8705	0.0749
141.	1289.6479	0.0908
142.	1295.6116	1.3772
143.	1299.3871	0.0216
144.	1304.8911	0.8664
145.	1309.6174	0.0063
146.	1314.3595	1.4078
147.	1326.0057	0.4979
148.	1327.6376	0.4139
149.	1342.1202	2.4705
150.	1348.4567	0.1993
151.	1355.8900	0.2010
152.	1359.4126	0.3258
153.	1361.9616	1.0322
154.	1370.9342	2.7638

155.	1374.5216	1.0259
156.	1377.8006	1.7679
157.	1382.6289	0.5815
158.	1384.0162	1.1792
159.	1389.5476	0.6213
160.	1399.6116	4.4997
161.	1408.8467	0.4641
162.	1414.6430	3.8920
163.	1425.3240	2.4689
164.	1431.2926	1.5375
165.	1444.1846	2.5527
166.	1449.9804	2.6494
167.	1464.6346	0.4704
168.	1471.2295	2.8463
169.	1478.0520	0.2408
170.	1494.4059	5.5921
171.	1519.3012	2.1637
172.	1532.0634	0.1215
173.	1533.4701	0.4612
174.	1542.8558	1.1232
175.	1545.5482	0.2338
176.	1548.3742	0.2310
177.	1566.9148	23.7835
178.	1572.5924	0.3383
179.	1575.1459	22.3463
180.	1578.6816	1.0144
181.	1591.6709	3.7268
182.	1603.2560	0.6339
183.	1613.5571	2.2536
184.	1616.9936	0.0308
185.	1627.0598	0.0513
186.	1630.5144	0.1764
187.	1631.8557	0.5144
188.	1646.1933	0.4751
189.	1650.8458	0.6683

Table S3. Natural Bond Orbital (NBO) donor–acceptor interactions for C24, C54, P51, and O65 computed using DFT at the ωB97X−D/6−31G(d) level of theory.

S. No.	Donor NBO (i)	Acceptor NBO (j)	E^2	E(j) - E(i)	F(i,j)
1.	BD(1)C24-C54	RY*(1)O65	0.55	2.83	0.035
2.	BD(1)C24-C54	RY*(4)O65	0.88	2.6	0.043
3.	BD(1)C24-C54	BD*(1)C24-O65	1.22	1.04	0.032
4.	BD(1)C24-C54	BD*(1)C54-O65	1.22	1.04	0.032
5.	BD(1)C24-O65	RY*(6)C54	0.89	2.87	0.046
6.	BD(1)C24-O65	BD*(1)C24-C54	3.11	1.24	0.056
7.	BD(1)C24-O65	BD*(1)C54-O65	10.77	1.1	0.098
8.	BD(1)C54-O65	RY*(6)C24	0.89	2.87	0.046
9.	BD(1)C54-O65	BD*(1)C24-C54	3.12	1.24	0.056
10.	BD(1)C54-O65	BD*(1)C24-O65	10.77	1.1	0.098
11.	CR(1)C24	BD*(1)C54-O65	1.55	10.55	0.117
12.	CR(1)C54	BD*(1)C24-O65	1.55	10.55	0.117
13.	CR(1)O65	RY*(1)C24	1.02	20.31	0.129
14.	CR(1)O65	RY*(5)C24	0.68	20.15	0.104
15.	CR(1)O65	RY*(1)C54	1.02	20.31	0.129
16.	CR(1)O65	RY*(5)C54	0.68	20.15	0.104
17.	CR(1)O65	BD*(1)C24-O65	0.74	19.37	0.11
18.	CR(1)O65	BD*(1)C54-O65	0.74	19.37	0.11
19.	LP(1)O65	RY*(4)C24	1.69	2.57	0.06
20.	LP(1)O65	RY*(4)C54	1.69	2.57	0.06
21.	LP(2)O65	RY*(1)C24	3	2.09	0.072
22.	LP(2)O65	RY*(1)C54	3	2.09	0.072
23.	LP(2)O65	BD*(1)C24-O65	1.24	1.16	0.034
24.	LP(2)O65	BD*(1)C54-O65	1.24	1.16	0.034
25.	BD(1)C24-O65	LP*(1)P61	0.9	0.82	0.029
26.	BD(1)C24-O65	RY*(5)P61	0.25	1.89	0.02
27.	BD(1)C24-O65	RY*(7)P61	0.1	1.62	0.012
28.	BD(1)C24-O65	RY*(8)P61	0.14	2.23	0.016
29.	BD(1)C54-O65	LP*(1)P61	0.9	0.82	0.029
30.	BD(1)C54-O65	RY*(5)P61	0.25	1.89	0.02
31.	BD(1)C54-O65	RY*(7)P61	0.1	1.62	0.012
32.	BD(1)C54-O65	RY*(8)P61	0.14	2.23	0.016
33.	CR(1)O65	LP*(1)P61	2.82	19.09	0.25
34.	CR(1)O65	RY*(7)P61	0.14	19.89	0.047
35.	CR(1)O65	RY*(8) P61	0.05	20.5	0.029
36.	LP(1)O65	RY*(4) P61	0.17	1.47	0.014
37.	LP(1)O65	RY*(6) P61	0.15	1.51	0.013
38.	LP(2)O65	LP*(1) P61	54.65	0.88	0.228
39.	LP(2)O65	RY*(7) P61	0.15	1.68	0.014
40.	LP(2)O65	RY*(8) P61	0.54	2.29	0.032
41.	LP(2)O65	RY*(9) P61	0.59	2.53	0.035

42.	LP(2)O65	RY*(10)P61	0.3	4.39	0.033
43.	CR(2)P61	BD*(1)C24-O65	0.2	8.16	0.037
44.	CR(2)P61	BD*(1)C54-O65	0.2	8.16	0.037
45.	CR(5)P61	BD*(1)C24-O65	0.11	5.13	0.021
46.	CR(5)P61	BD*(1)C54-O65	0.11	5.13	0.021
47.	LP*(1)P61	RY*(1)O65	2.14	2.06	0.105
48.	LP*(1)P61	RY*(4)O65	0.07	1.84	0.018
49.	LP*(1)P61	RY*(6)O65	0.13	2.43	0.028
50.	LP*(1)P61	RY*(10)O65	0.09	3.9	0.03
51.	LP*(1)P61	BD*(1)C24-O65	5.61	0.28	0.059
52.	LP*(1)P61	BD*(1)C54-O65	5.61	0.28	0.059

Table S4. The *in-silico* toxicity report

Classification Domain	Target Endpoint	Prediction	Probability
**Organ Toxicity**	Hepatotoxicity	Inactive	0.64
	Neurotoxicity	Inactive	0.88
	Nephrotoxicity	Inactive	0.51
	Respiratory toxicity	Active	0.55
	Cardiotoxicity	Inactive	0.77
**Toxicity End Points**	Carcinogenicity	Inactive	0.58
	Immunotoxicity	Inactive	0.84
	Mutagenicity	Active	0.66
	Cytotoxicity	Inactive	0.78
	BBB-barrier Penetration	Active	0.78
	Ecotoxicity	Inactive	0.54
	Clinical toxicity	Inactive	0.64
	Nutritional toxicity	Inactive	0.52
**Tox21-Nuclear Receptor Signalling Pathways**	Aryl hydrocarbon Receptor (AhR)	Inactive	0.75
	Androgen Receptor (AR)	Inactive	0.95
	Androgen Receptor Ligand Binding Domain	Inactive	0.93
	Aromatase	Inactive	0.88
	Estrogen Receptor Alpha (ER)	Inactive	0.71
	Estrogen Receptor Ligand Binding Domain	Inactive	0.86
	Peroxisome Proliferator Activated Receptor Gamma	Inactive	0.95
**Tox21-Stress Response Pathways**	Nuclear factor (erythroidderived -2)-like 2/ARE	Inactive	0.82
	Heat shock factor response element (HSE)	Inactive	0.82
	Mitochondrial Membrane Potential (MMP)	Inactive	0.72
	Phosphoprotein (Tumor Suppressor) p53	Inactive	0.84

	ATPase family AAA domain-containing protein 5	Inactive	0.95
**Molecular Initiating Events**	Thyroid hormone receptor alpha (THR)	Inactive	0.8
	Thyroid hormone receptor beta (THRβ)	Inactive	0.8
	Transthyretin (TTR)	Active	0.69
	Ryanodine receptor (RYR)	Inactive	0.85
	GABA receptor (GABAR)	Inactive	0.68
	Glutamate N-methyl-D-aspartate receptor	Inactive	0.84
	α-amino-3-hydroxy-5-methyl-4-isoxazolepropionate	Inactive	0.99
	Kainate receptor (KAR)	Inactive	1
	Acetylcholinesterase (AChE)	Inactive	0.65
	Constitutive androstane receptor (CAR)	Inactive	0.98
	Pregnane X receptor (PXR)	Active	0.57
	NADH-quinone oxidoreductase (NADHOX)	Inactive	0.85
	Voltage gated sodium channel (VGSC)	Inactive	0.86
	Na+/I− symporter (NIS)	Inactive	0.86
**Metabolism (Cytochromes)**	Cytochrome CYP1A2	Inactive	0.78
	Cytochrome CYP2C19	Inactive	0.77
	Cytochrome CYP2C9	Inactive	0.5
	Cytochrome CYP2D6	Inactive	0.82
	Cytochrome CYP3A4	Inactive	0.82
	Cytochrome CYP2E1	Inactive	0.97
